# Differential contributions of fatigue‐induced strength loss and slowing of angular velocity to power loss following repeated maximal shortening contractions

**DOI:** 10.14814/phy2.14362

**Published:** 2020-02-07

**Authors:** Ryota Akagi, Avery Hinks, Brooke Davidson, Geoffrey A. Power

**Affiliations:** ^1^ College of Systems Engineering and Science Shibaura Institute of Technology Saitama Japan; ^2^ Department of Human Health and Nutritional Sciences College of Biological Science University of Guelph Guelph ON Canada

**Keywords:** dorsiflexion, isometric contraction, isotonic contraction, neuromuscular fatigue

## Abstract

The purpose of this study was to investigate the relationship between fatigue‐induced reductions in isometric torque and isotonic power and to quantify the extent to which the decreases in angular velocity and dynamic torque can explain the power loss immediately following an isotonic fatiguing task and throughout recovery in seven young males and six young females. All measurements were performed with both legs. For dorsiflexion, fatigue‐related time‐course changes in isometric maximal voluntary contraction (MVC) torque, angular velocity, dynamic torque, and power production following repeated maximal isotonic contractions (load: 20% MVC) were investigated before, immediately after, and 1, 2, 5 and 10 min after a fatiguing task. There were no relationships between the fatigue‐related reductions in isometric MVC torque and peak power at any timepoint, suggesting that fatigue‐induced reductions in isometric MVC torque does not entirely reflect fatigue‐induced changes in dynamic performance. The relative contribution of fatigue‐related reduction in dynamic torque on power loss was greater immediately following the task, and lower throughout recovery than the corresponding decrease in angular velocity. Thus, power loss immediately following the task was more strongly related to the decline in dynamic torque; however, this relationship shifted throughout recovery to a greater dependence on slowing of angular velocity for power loss.

## Graphical abstract

The current study used multiplication of the standard regression coefficient and Pearson's product–moment correlation coefficient to tease out the relative contributions of angular velocity and dynamic torque to power production immediately following a maximal dynamic fatiguing task and throughout recovery. Here, we show for the first time that the relative contributions of fatigue‐induced slowing of angular velocity and decrease in dynamic torque to power loss vary depending on the timepoints from immediately following the fatiguing task to throughout recovery.

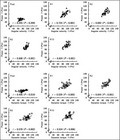

## 1 INTRODUCTION

Fatigue can be defined as any exercise‐induced reduction in the ability to generate force or power, regardless of whether or not the task can be sustained (Gandevia, 2001), and can be quantified as time‐course changes in maximum voluntary contraction (MVC) torque (or force), and/or power during and following a fatiguing task (Lanning, Power, Christie, & Dalton, 2017; Lee et al., 2017; Senefeld, Pereira, Elliott, Yoon, & Hunter, 2018; Thompson, Conchola, & Stock, 2015; Wallace, Power, Rice, & Dalton, 2016). Optimal performance of dynamic contractions requires not only muscle force production but also a velocity component, as such, relying solely on measurements of isometric MVCs to reflect fatigue‐induced changes in neuromuscular function, may be inappropriate, especially when making inferences on fatigue‐induced changes in dynamic performance (Cheng & Rice, 2009; Dalton, Power, Vandervoort, & Rice, 2010). However, researchers continue to use isometric MVC torque to examine neuromuscular fatigue and/or recovery because testing can be performed with basic equipment and fewer extraneous factors (Cheng & Rice, 2005), and the measurement of isometric MVC torque is easy to combine with the interpolated twitch technique (ITT) to determine central and peripheral fatigue. A recent study (Krüger et al., 2019) found that isometric assessment of fatigue (isometric MVC force) is not interchangeable with dynamic assessment of fatigue (maximal power and torque); however, they used a recumbent cycling ergometer to compare the isometric and dynamic measures despite the fact that power delivered to the pedals is produced by muscles that span the ankle, knee and hip during cycling (Barratt, Martin, Elmer, & Korff, 2016; Martin & Brown, 2009). In other words, it is unclear whether this finding can also be applied to a specific muscle group regardless of timepoints during recovery. Considering the versatility of using isometric MVCs to assess neuromuscular fatigue, and to bring some consensus to the literature, it is important to clarify the relationship between the changes in power and isometric MVC torque immediately following a fatiguing task and throughout recovery by examining a specific muscle group.

Isokinetic contractions are used frequently to assess power (Senefeld et al., 2018; Thompson et al., 2015). However, everyday movements are characterized by ballistic sinusoidal changes in velocity with constant loads (Cairns, Knicker, Thompson, & Sjogaard, 2005) rather than movements of constant velocity. In other words, isotonic contractions in which the load is held constant but the velocity can vary are considered more relevant to normal voluntary activity than isokinetic. As suggested previously (Cheng & Rice, 2009), fatigue‐induced power loss is only partially explained by the corresponding reduction in isometric MVC torque, and thus, assessments of shortening velocity are useful in identifying the underlying factors contributing performance fatigability (Cheng & Rice, 2009; Dalton et al., 2010). Furthermore, considering that fatigue‐induced power loss is partly due to an increase in curvature of the force‐velocity relationship of a muscle induced by fatigue (Jones, 2010; Kristensen, Nielsen, Pedersen, & Overgaard, 2019), a fatigue‐induced decline in dynamic torque can also be an important parameter for understanding the mechanism of neuromuscular fatigue and recovery. Recently, a positive relationship has been reported between fatigue‐related decreases in angular velocity, dynamic torque and power loss (Lanning et al., 2017; Wallace et al., 2016) using isotonic contractions of the plantar flexors; however, the difference in the extent to which the reductions in angular velocity and dynamic torque can explain power loss following a fatiguing task has not been statistically quantified. Additionally, the corresponding relationships of angular velocity or dynamic torque with power have not been investigated throughout recovery, given these two measures recover at different rates, the relative contributions to fatigue‐induced power loss is unclear. Thus, assessing power loss following a fatiguing task is important for accurately understanding the etiology of neuromuscular fatigue and recovery, and the aforementioned relative contributions of angular velocity and dynamic torque to power can vary depending on the elapsed time after completion of task. Therefore, it is essential to clarify relative contributions of angular velocity and dynamic torque on power production at task failure and throughout recovery.

The purpose of the current study was to investigate the relationship between fatigue‐induced reductions in isometric MVC torque and isotonic power, and to quantify the extent to which the decreases in angular velocity and dynamic torque can explain the power loss immediately following an isotonic fatiguing task and throughout recovery. In the current study, the model we used was dorsiflexion because complete voluntary activation (VA) is often observed with this muscle group (Baudry, Klass, Pasquet, & Duchateau, 2007; Chen & Power, 2019; Cheng & Rice, 2009) during isometric MVCs, and we expected “near maximal activation” during maximal effort dynamic contractions.

## 2 MATERIALS AND METHODS

### 2.1 Participants

Seven males (age: 27 ± 7 yr, height: 178.5 ± 6.3 cm, body mass: 78.8 ± 11.0 kg; mean ± standard deviation (*SD*)) and six females (age: 22 ± 3 yr, height: 169.7 ± 8.3 cm, body mass: 63.2 ± 4.2 kg; mean ± *SD*) participated in this study and all measurements were performed with both legs. Participants were free of cardiovascular and neuromuscular diseases, recreationally active, and did not perform any specific training regarding ankle dorsiflexion. This study was approved by the Local Research Ethics Board.

### 2.2 Experimental arrangements and procedures

#### 2.2.1 Set up

Measurements were performed using one leg on the first day and using the other leg on the second day. Participants were seated on a HUMAC NORM dynamometer (CSMi Medical Solutions, USA) with the hip at 70°, knee flexed at 40°, the ankle at 40° of plantar flexion (anatomical positions = 0°), and medial malleolus aligned with the dynamometer's axis of rotation. The pelvis and ankle were secured on the seat and a foot pedal adapter, respectively, with a seat belt and/or non‐elastic straps.

A standard clinical bar electrode (interelectrode distance of 30 mm; Empi, St Paul, MN, USA) coated in conductive gel was placed over the head of the fibula to percutaneously stimulate the deep fibular nerve. Peripheral nerve stimulation was delivered using constant current stimulator (single pulse; pulse width: 200μs; 400V; model DS7AH, Digitimer, UK). Stimulus intensity was initially increased until plateau in the twitch torque was reached. Supramaximal stimulus intensity was set at the electrical current calculated by multiplying the stimulus intensity by 1.4 for experimental measurements in order to ensure maximal muscle activation.

#### 2.2.2 Static and dynamic contractions

Prior to the fatiguing task, a supramaximal doublet stimulation at 100 Hz was interpolated to obtain doublet peak torque. Afterward, the participants were instructed to perform a 3‐s isometric dorsiflexion MVCs twice with a 3‐min rest interval, encouragement and visual feedback were provided in the same way every time, and the highest torque value was used as “Pre.” On the second performance of isometric MVC, VA was assessed using the ITT. Two supramaximal doublet stimulations at 100 Hz were interpolated approximately 2 s after the beginning and end of contraction, respectively, for VA assessment using the following formula: VA (%) = [1 − (interpolated twitch torque/control twitch torque)] × 100. Activation value of 95% VA or higher was deemed “near maximal” (for details please see Chen & Power, 2019), and all participants met this condition before the fatiguing task. The reliability and validity of the ITT were ensured elsewhere (Behm, St‐Pierre, & Perez, 1996). Afterward, the participants were instructed to perform maximal effort isotonic dorsiflexions with a load set to 20%MVC three times every 3–4 s, from 40° of plantar flexion to 0°. Peak power was calculated using the product of angular velocity and torque in real time. The average of the three values of peak power was used as “Pre,” and angular velocity and dynamic torque at instantaneous peak power were reported, respectively. During each contraction, joint torque, angular velocity, and power data were displayed as waveforms on a computer monitor and placed 1.5 m in front of the participants. The torque, power, and angular velocity data were stored at a sampling frequency of 2,000 Hz on a personal computer using LabChart software (v8.1.11, ADInstruments) after the A/D conversion (PowerLab16/35, ADInstruments) and were smoothed using a ten‐point moving average. Only during offline analysis of doublet peak torque and VA, the torque data were low‐pass‐filtered at 500 Hz instead of the aforementioned smoothing.

#### 2.2.3 Fatiguing task and recovery

For the fatiguing task, the participants repeated maximal effort 20%MVC isotonic dorsiflexions (Power, Dalton, Rice, & Vandervoort, 2011) over the 40° range of motion until peak power fell below 60% of Pre twice in a row. Immediately after the fatiguing task (Post), doublet peak torque and isometric MVC torque with VA were determined once. There was approximately 3–5 s delay between the end of the fatiguing task and the doublet stimulation. Regarding the peak power and the angular velocity and dynamic torque at instantaneous peak power, the data of the last contraction of the fatiguing task were used as the values at Post.

Throughout recovery (i.e., 1 [R1], 2 [R2], 5 [R5] and 10 min [R10] following task failure), doublet peak torque was obtained once, and then, an MVC with ITT and two maximal effort isotonic contractions were performed. The highest peak power and the related angular velocity and dynamic torque were evaluated at each timepoint.

### 2.3 Statistical analyses

First, the values relative to Pre were calculated for each parameter and we confirmed that there were no effects of sex or leg dominance (the preferred kicking leg) on the relative values of each parameter at any timepoint using a three way repeated measures analysis of variance (ANOVA). Therefore, all of the data were pooled before performing the following analyses (i.e., total sample size: *n* = 26). Considering the experimental design, a priori sample size estimation was performed in two conditions using G*Power software package (Version 3.1.9.4, Kiel University) before other statistical analyses. One was for a correlation calculation (Statistical test = Correlation: Point biserial model; Tail(s) = Two; Effect size |ρ| = 0.5; *α* err prob = 0.05; Power (1 − *β* err prob) = 0.80). The other was for a one‐way repeated measures ANOVA (Statistical test = ANOVA: Repeated Measures, within factors; Effect size *f* = .25; *α* err prob = 0.05; Power (1 − *β* err prob) = 0.80; Number of groups = 1; Number of measurements = 6; Corr among rep measures = 0.5; Nonsphericity correction e = 1). The total sample size (*n* = 26) was satisfied with the above two conditions.

A one‐way ANOVA with a within‐group factor (timepoint [Pre, Post and R1–R10]) was used to evaluate time‐course changes in each parameter. Considering many timepoints, when a significant main effect was detected, a multiple comparison was performed using a paired *t*‐test with Holm‐Bonferroni correction.

To compare the difference in the time‐course change between the values of power and isometric MVC torque, or between power, angular velocity and dynamic torque relative to the baseline, a two‐way ANOVA with within‐group factors (timepoint [Post and R1–R10] and parameter [the former comparison: power and isometric MVC torque; the latter comparison: power, angular velocity and dynamic torque]) was used. When a significant interaction was detected, Bonferroni multiple comparisons were performed to examine the differences between the relative values of the corresponding parameters at each timepoint.

To investigate the relationship between the time‐course changes in power and isometric torque, Pearson's product–moment correlation coefficients between the relative values of isometric torque and power at each timepoint were calculated. To quantify the relative contributions of reductions in angular velocity and dynamic torque on power loss after the fatiguing task and their changes over time, the following procedure was performed at each timepoint according to previous studies (Akagi et al., 2018; Miyatani, Kanehisa, Ito, Kawakami, & Fukunaga, 2004): after calculating Pearson's product–moment correlation coefficients between the relative values of angular velocity or dynamic torque and power, a stepwise multiple regression analysis using the relative values of angular velocity and dynamic torque as the independent variables and that of power as the dependent variable was performed. When both were selected as significant contributors, the standard regression coefficients in the multiple regression equation and the Pearson's product–moment correlation coefficients were multiplied. These values show the extent to which the reductions in angular velocity and dynamic torque can explain power loss following the isotonic fatiguing task.

Data in the text and figures are presented as means ± *SD*s. Statistical significance was set to *p* < .05. For the main effect and interaction of the one‐way or two‐way ANOVAs, *η* ^2^ was calculated as an index of the effect size. In addition, when a paired *t*‐test with Holm‐Bonferroni correction revealed a significant difference between the two values, Cohen's *d* was calculated as another index of the effect size. The values of *η* ^2^ or *d* were interpreted as 0.01 ≤ *η* ^2^ < 0.06 or 0.20 ≤ *d* < 0.50 for small, 0.06 ≤ *η* ^2^ < 0.14 or 0.50 ≤ *d* < 0.80 for medium, and 0.14 ≤ *η* ^2^ or 0.80 ≤ *d* for large effects, respectively (Cohen, 1988). In accordance with a previous study (Ema, Suzuki, Kawaguchi, Kawaguchi, & Akagi 2018), we considered the main effects, interactions or differences to be substantial if both effect size was ≥medium (i.e., *η* ^2^ ≥ 0.06 or *d* ≥ 0.50) and *p* < .05. Statistical analyses were performed using statistical analysis software (SPSS 25.0, IBM) and an Excel calculator for Holm‐Bonferroni correction (Gaetano, 2018).

## 3 RESULTS

The number of repetitions during the fatiguing task was 39 ± 20 times, ranging from 9–103.

**Figure 1:**
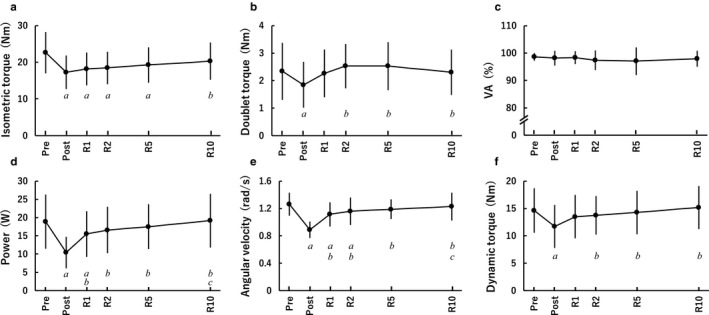
Peak torque during isometric maximal voluntary contraction (a), doublet peak torque (b), voluntary activation (c), peak power during maximal effort isotonic contraction (d), and angular velocity (e) and dynamic torque (f) at instantaneous peak power before (Pre) and immediately (Post), 1 min (R1), 2 min (R2), 5 min (R5), and 10 min (R10) after the fatiguing task (*n* = 26). In these figures, we considered the main effects or differences to be substantial if both effect size ≥ medium and *p* < .05. Except for voluntary contraction, there were substantial main effects of timepoints for each parameter. *a*, *b*, and *c* indicate substantial differences from Pre, Post, and R1, respectively. Data are presented as means ± *SD*

Figure 1a–f displays the time‐course changes in isometric MVC torque, doublet torque, VA and dynamic parameters, with a substantial main effect of timepoint for all measures (isometric MVC torque: *F*[2.804,70.100] = 37.098, *p* < .001, *η* ^2^ = 0.60; doublet torque: *F*[3.233,80.813] = 11.062, *p* < .001, *η* ^2^ = 0.31; power: *F*[3.071,76.781] = 106.522, *p* < .001, *η* ^2^ = 0.81; angular velocity: *F*[5,125] = 70.656, *p* < .001, *η* ^2^ = 0.74; dynamic torque: *F*[5,125] = 41.532, *p* < .001, *η* ^2^ = 0.62) except for VA. Immediately following the fatiguing task, isometric MVC torque was reduced by 23.6% (*p* < .001, *d* = 1.04), and did not recover substantially by R5 (*p* < .001, *d* ≥ 0.64). There was a significant reduction in isometric torque from Pre to R10 (*p* = .006), but it was not substantial (*d* = 0.43). Doublet torque was reduced by 21.1% immediately after the fatiguing task (*p* = .027, *d* = 0.53) and recovered by R1. No main effect was found (*F*[3.788,94.709] = 1.086, *p* = .366, *η* ^2^ = 0.04) for VA, indicating “near maximal activation” throughout. Peak power was reduced by 44.9% immediately following the fatiguing task (*p* < .001, *d* = 1.40) and recovered by R2. Angular velocity and dynamic torque were decreased by 29.8% (*p* < .001, *d* = 2.60) and 19.9% (*p* < .001, *d* = 0.73) immediately following the fatiguing task, and recovered by R5 and R1, respectively.

**Figure 2:**
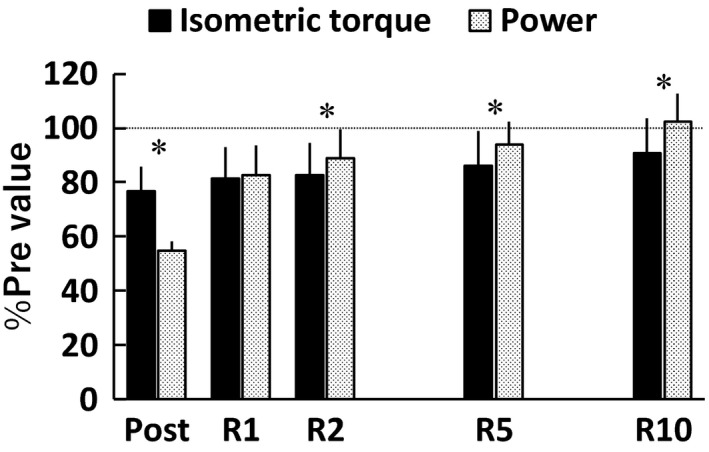
The values relative to the baseline values (%Pre) of peak torque during isometric maximal voluntary contraction and peak power during maximal effort isotonic contraction immediately (Post), 1 min (R1), 2 min (R2), 5 min (R5), and 10 min (R10) after the fatiguing task (*n* = 26). In this figure, we considered the main effects or differences to be substantial if both effect size ≥ medium and *p* < .05. There was a substantial timepoint × parameter interaction. * indicates a substantial difference at the same timepoint. Data are presented as means ± *SD*

**Figure 3:**
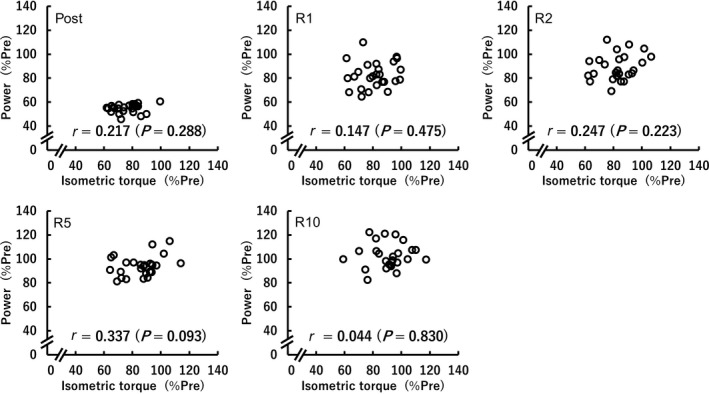
Relationships between the values relative to the baseline values (%Pre) of peak torque during isometric maximal voluntary contraction and peak power during maximal effort isotonic contraction at each timepoint (*n* = 26)

Regarding the fatigue‐induced relative reductions in isometric MVC torque and peak power during maximal effort isotonic contraction (Figure 2), there was a substantial timepoint × parameter interaction (*F*[4,100] = 70.170, *p* < .001, *η* ^2^ = 0.17). At Post, the reduction in isotonic power was substantially greater than that of isometric MVC torque (*p* < .001, *d* = 3.15). In contrast, isotonic power recovered faster than isometric MVC torque at R2–R10 (*p* = .001–0.031, *d* = 0.55–0.98). At each timepoint, there were no significant relationships between the relative reductions in isometric MVC torque and isotonic power (*r* = .044–.337, *p* = .093–0.830) (Figure 3).

**Figure 4:**
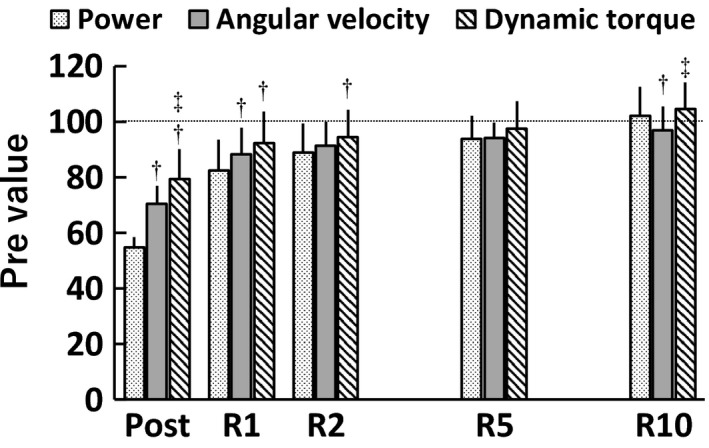
The values relative to the baseline values (%Pre) of peak power during maximal effort isotonic contraction and angular velocity and dynamic torque at instantaneous peak power immediately (Post), 1 min (R1), 2 min (R2), 5 min (R5), and 10 min (R10) after the fatiguing task (*n* = 26). In this figure, we considered the main effects or differences to be substantial if both effect size ≥ medium and *p* < .05. There was a substantial timepoint × parameter interaction. † and ‡ indicate substantial differences from power and angular velocity, respectively, at the same timepoint. Data are presented as means ± *SD*

**Figure 5:**
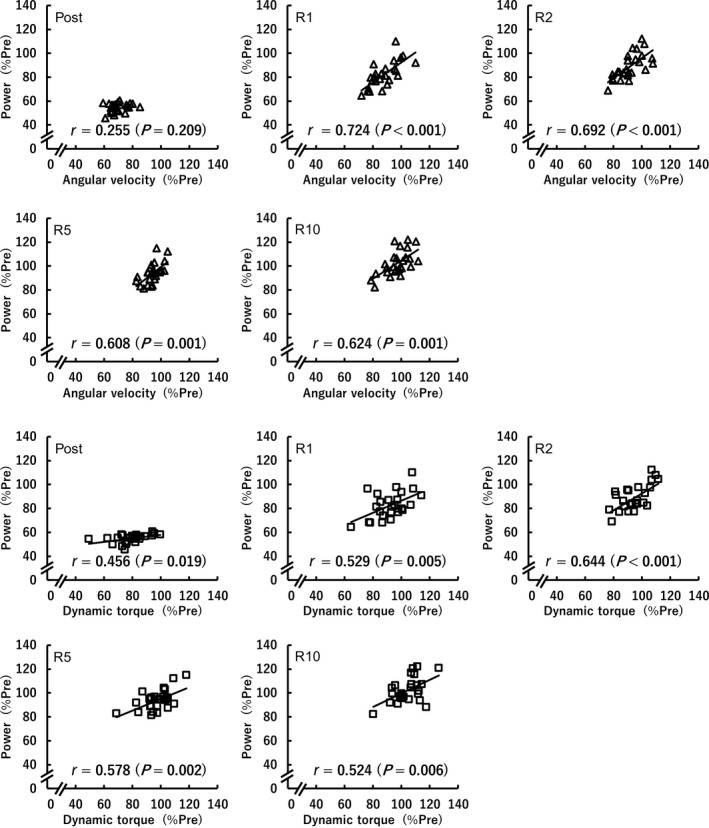
Relationships between the values relative to the baseline values (%Pre) of angular velocity (△) or dynamic torque (□) at instantaneous peak power and peak power during maximal effort isotonic contraction at each timepoint (*n* = 26)

A timepoint × parameter interaction (*F*[5.623,140.581] = 19.236, *p* < .001, *η* ^2^ = 0.06) was found for the relative values of dynamic parameters (Figure 4). Following the fatiguing task, compared to angular velocity, power was substantially lower at Post and R1 (*p* ≤ .003, *d* = 0.56–3.04) and recovered more quickly by R10 (*p* = .012, *d* = 0.55), and dynamic torque was substantially higher at Post (*p* = .021, *d* = 1.00) and recovered more quickly by R10 (*p* = .032, *d* = 0.82). The substantial differences between the relative values of power and dynamic torque were found at Post, R1 and R2 (power < dynamic torque; *p* ≤ .007, *d* = 0.57–3.04). Here, the relative contributions of fatigue‐induced reductions in angular velocity and dynamic torque to power loss were quantified using the aforementioned method (Akagi et al., 2018; Miyatani et al., 2004). Each of the relative values of angular velocity (*r* = .608–.724, *p* ≤ .001) and dynamic torque (*r* = .456–.644, *p* ≤ .019) (Figure 5) were positively correlated with that of power at each timepoint except for the relationship between the relative values of angular velocity and power at Post (*r* = .255, *p* = .209). The stepwise multiple regression analysis revealed that the relative values of angular velocity (Post: *β* = .751; R1: *β* = .708; R2: *β* = .597; R5: *β* = .624; R10: *β* = .721) and dynamic torque (Post: *β* = .879; R1: *β* = .507; R2: *β* = .538; R5: *β* = .595; R10: *β* = .635) were both significant contributors for estimating power at each timepoint (*p* < .001). As a result of multiplication of the standard regression coefficient and Pearson's product–moment correlation coefficient, the relative contributions of reductions in angular velocity and dynamic torque on power loss were 19.1% and 40.1% at Post, 51.3% and 26.8% at R1, 41.3% and 34.6% at R2, 37.9% and 34.4% at R5, and 45.0% and 33.3% at R10, respectively. Briefly, reduction in dynamic torque had a greater influence than slowing of angular velocity on power loss immediately following the fatiguing task; however, that relationship shifted throughout recovery to a greater dependence on slowing of angular velocity for power loss.

## 4 DISCUSSION

The purpose of the present study was twofold: (a) to investigate the relationship between fatigue‐induced reductions in isometric MVC torque and isotonic power, and (b) to statistically identify the relative contributions of dynamic torque and angular velocity to power production immediately following a fatiguing task and throughout recovery. Regarding purpose (a), relationships between the fatigue‐related reductions in isometric MVC torque and peak power were not found at any timepoint. For purpose (b), we found that the relative contribution of fatigue‐induced reduction in dynamic torque to power loss was more than twice that of fatigue‐induced slowing of angular velocity immediately following the fatiguing task, but that relation shifted throughout the recovery phase to the slowing of angular velocity better predicting power loss. Previously, it was reported that reductions in angular velocity was the key contributor to isotonic power loss (Cheng & Rice, 2009; Dalton et al., 2010); however, given the analysis used by those authors, it was not possible to tease out the relative contributions. Using multiplication of the standard regression coefficients in the multiple regression equation and the Pearson's product–moment correlation coefficients, we show for the first time that the relative contributions of fatigue‐induced slowing of angular velocity and decrease in dynamic torque to power loss vary depending on the timepoints from immediately following the fatiguing task to throughout recovery.

The fatigue‐induced reduction in isometric MVC torque remained until R5 (Figure 1a), while isotonic power recovered by R2 (Figure 1d)). Thus, isotonic power recovered sooner than isometric MVC torque (Figure 2), which is consistent with previous findings (Cheng & Rice, 2005; Dalton, Power, Paturel, & Rice, 2015). Moreover, in line with previous studies (Cheng & Rice, 2005; Jones, Ruiter, & Haan, 2006), the fatigue induced reduction in isometric MVC torque was less than the reduction in power (Figure 2), indicating a greater impairment in dynamic than static performance. Thus, the time‐course change in isometric MVC torque was not consistent with that in isotonic power following repeated isotonic contractions. Consequently, no significant correlations between the relative values of isometric MVC torque and isotonic power were found (Figure 3). Some previous studies (Cheng & Rice, 2009; Dalton et al., 2010; Krüger et al., 2019) described that fatigue‐induced reductions in isometric MVC torque do not entirely reflect fatigue‐induced changes in the muscle, especially as related to dynamic performance. A recent study (Krüger et al., 2019) also suggested that isometric assessment of fatigue is not interchangeable with dynamic assessment of fatigue when cycling. Based on the present findings, we can emphasize that this idea is applicable not only immediately following the fatiguing task but also throughout recovery, regardless of the timepoint. It is indicated that fatigue‐related torque decrease is related to impairments in excitation‐contraction coupling (Klass, Guissard, & Duchateau, 2004), namely, lower myoplasmic concentrations of Ca^2+^ and/or decreased Ca^2+^ sensitivity caused by fatigue (MacIntosh, Holash, & Renaud, 2012). Moreover, lower levels of ATP and high concentrations of Pi during fatigue are considered to influence force production by reducing the energy charge, decreasing the specific force per cross‐bridge and the rate of cross‐bridge dissociation (Edwards, Hill, & Jones, 1975; Jones, Turner, McIntyre, & Newham, 2009), likely resulting in the corresponding decrease in isometric torque. On the other hand, increased ADP is suggested to contribute to fatigue‐related reduction in angular velocity (Krüger et al., 2019) owing to a slowing of maximal velocity (Westerblad, Dahlstedt, & Lännergren, 1998; Westerblad & Lännergren, 1995). Considering power is dependent not only torque but also velocity, it is reasonable to consider that power is affected by a combination of many of the above mentioned factors. Thus, the main factors impairing the ability to generate isometric torque appear to be different from those of isotonic contractions as a previous study indicated (Cheng & Rice, 2005), and this difference is a reason for the discrepancy between the time‐course changes in isometric MVC torque and power following repeated isotonic contractions and no relationships between them at any timepoint.

In the present study, the time‐course changes in power and the value relative to the baseline were not always consistent with those in angular velocity and dynamic torque (Figures 1d–f and 4) despite the fact that both the fatigue‐related reductions in angular velocity and dynamic torque have been reported to impair power production (Lanning et al., 2017; Wallace et al., 2016). Briefly, power recovered faster than angular velocity and slower than dynamic torque. Consequently, the relative contributions of reductions in angular velocity and dynamic torque on power loss varied depending on the timepoint: The relative contribution of fatigue‐related reduction in dynamic torque on power loss was greater immediately following the task, and lower throughout recovery than the corresponding decrease in angular velocity. These results appear to be affected by the increase in the curvature of the force‐velocity relationship due to fatigue, influencing power loss (Jones, 2010; Kristensen et al., 2019).

We used a 20%MVC isotonic load as the fatiguing task in the present study. As a result, both isometric MVC torque (Figure 2) and dynamic torque (Figure 4) decreased to about 80% of baseline values immediately following the fatiguing task, but the time‐course change in dynamic torque (Figure 1f) did not correspond that in isometric MVC torque (Figure 1a). Furthermore, despite no relationships between the fatigue‐related reductions in isometric MVC torque and isotonic peak power at any timepoint (Figure 3), the fatigue‐induced decline in dynamic torque still had a major influence on power loss (Figure 5). In a study of Krüger et al. (2019), they expected the mechanisms responsible for reduction and recovery of isometric and dynamic torque to be the same before their experiment, but found that there were differences in the fatigue‐induced time‐course changes in isometric and dynamic torque in several conditions. These results were similar to the current results (Figure 1a and f). Therefore, it is difficult to clearly identify the reason for the fatigue‐induced reduction in dynamic torque; however, there was a clear difference in the relationship with power loss between reductions in isometric MVC torque (Figure 3) and dynamic torque (Figure 5). Considering this difference together with the same degree of reductions in isometric MVC torque and dynamic torque immediately after the fatiguing task, it is expected that the current findings do not change, regardless of the load of isotonic contraction requiring maximal unconstrained velocity. Further studies using different loads of the fatiguing task are required to strengthen the findings of the current study.

Immediately following the fatiguing task, compared with the correlation coefficients of power loss with fatigue‐induced reductions in angular velocity (*r* = .91–.94) or dynamic torque (*r* = .87–.96) of previous studies (Lanning et al., 2017; Wallace et al., 2016), those of the current study (*r* = .255 for angular velocity and *r* = .456 for dynamic torque) were very low (Figure 5). This discrepancy is likely owing to the task dependent nature of fatigue. In the previous studies, all participants continued the fatiguing task the same number of times (200 times in Lanning et al., 2017; 50 times in Wallace et al., 2016). However, in the current study, the participants repeated maximal effort 20%MVC isotonic dorsiflexions until peak power fell below 60% of Pre twice in a row. Therefore, as each figure in these studies shows, individual variations in power were obviously greater in the previous studies (Lanning et al., 2017; Wallace et al., 2016) than in the current study. The amount of variability of parameters affects the degree of correlation (Goodwin & Leech, 2006), and thus it is not surprising that the aforementioned discrepancy in the correlation coefficients between the studies. In addition, when performing a 2‐tailed test for difference in 2 coefficients of variance (CVs) (Zar, 1999) to investigate the differences between individual variations in the values of angular velocity and dynamic torque relative to the baseline, the CV of angular velocity (9.1%) was significantly lower than that of dynamic torque (13.7%) at Post (*p* < .05). Therefore, the greater individual variation in fatigue‐induced reduction in dynamic torque is suggested to result in the greater relative contribution on fatigue‐induced power loss (40.1%) compared to angular velocity (19.1%) at Post, which is in contrast to previous findings that evaluation of shortening velocity is useful in identifying the underlying factors contributing performance fatigability (Cheng & Rice, 2009; Dalton et al., 2010). On the other hand, throughout recovery, the relative contributions of reduction in angular velocity on power loss (R1: 51.3%, R2: 41.3%, R5: 37.9%, R10: 45.0%) was 1.10–1.91 times higher than those of reduction in dynamic torque (R1: 26.8%, R2: 34.6%, R5: 34.4%, R10: 33.3%), supporting the above previous findings.

The current study emphasizes the greater importance of joint angular velocity than dynamic torque for understanding muscle recovery except immediately after the fatiguing task. Isokinetic contractions, which maintains a constant angular velocity, have been used frequently to evaluate neuromuscular fatigue and recovery after dynamic contractions (Senefeld et al., 2018; Thompson et al., 2015). However, the result of a greater relative contribution of reduction in angular velocity than dynamic torque throughout recovery indicates that such contractions seem to be less appropriate to assess individual differences in recovery of skeletal muscles than isotonic contractions requiring maximal unconstrained velocity. Moreover, these data may indicate the utility of organizing exercise programs to improve fatigue resistance of contraction force and/or to promote fatigue recovery of contraction velocity for optimal performance in various sports and rehabilitation settings.

Fatigue depends on many peripheral and central factors (Maffiuletti et al., 2007). In accordance with previous studies (Burnley, Vanhatalo, & Jones, 2012; Pethick, Winter, & Burnley, 2018; Theurel & Lepers, 2008), we considered that doublet torque is an index of peripheral fatigue and VA is an index of central fatigue in the current study. For the dorsiflexors, complete VA is often observed during MVC (Baudry et al., 2007; Chen & Power, 2019; Cheng & Rice, 2009), and this study also found “near maximal activation” throughout (Figure 1c) as expected. Thus, it is suggested that voluntary drive was maintained and did not explain task failure in the current study. On the other hand, doublet torque substantially decreased immediately after the fatiguing task (Figure 1b) despite including the effect of post‐activation potentiation, likely due to impairments of excitation‐contraction coupling and cross‐bridge function (Cheng & Rice, 2009; Jones et al., 2006; Klass et al., 2004; Senefeld et al., 2018). Hence, fatigue was likely to be related to peripheral factors more closely than central factors in the current study—however, we cannot completely rule out the contribution of central fatigue.

## 5 CONCLUSIONS

The present study found no relationships between the fatigue‐related reductions in isometric MVC torque and isotonic peak power at any timepoint. These results emphasize the idea that fatigue‐induced reductions in isometric MVC torque does not entirely reflect fatigue‐induced changes in the muscle, especially as related to dynamic performance not only immediately following the fatiguing task but also throughout recovery regardless of the timepoint. Regarding dynamic parameters, the fatigue‐related time‐course changes were different among them, and the relative contribution of reduction in dynamic torque on power loss was higher immediately following the fatiguing task and lower throughout recovery than the corresponding decrease in angular velocity. Thus, it is suggested that power loss immediately following the task is more strongly related to decline in dynamic torque; however, this relationship shifts throughout recovery to a greater dependence on slowing of angular velocity for power‐loss.

## CONFLICT OF INTEREST

No conflict of interests, financial, or otherwise are declared by the authors.
